# Body mass index affects kidney transplant outcomes: A cohort study over 5 years using a steroid sparing protocol

**DOI:** 10.3389/fendo.2023.1106087

**Published:** 2023-02-09

**Authors:** Maria Irene Bellini, Emily Deurloo, Fabrizio Consorti, Paul Elliot Herbert

**Affiliations:** ^1^ Department of Surgery, Sapienza University, Rome, Italy; ^2^ Renal Transplant Department, Hammersmith Hospital, Imperial College National Health System (NHS) Trust, London, United Kingdom

**Keywords:** obesity, kidney transplant, body mass index, bariatric surgery, equity

## Abstract

**Background:**

There is controversy regarding the suitability of high body mass index (BMI) candidates accessing the transplant waitlist.

**Patients and methods:**

Observational study on consecutive kidney transplant recipients undergoing surgery between January 2014 and March 2016 at our center. Patients were stratified according to BMI. Survival outcomes and graft function were analyzed to investigate the effect of donor’s and recipient’s demographic characteristics.

**Results:**

396 kidney transplant recipients: 260 males, mean age 51.8 ± 15.9 years, followed up for a mean time of 5.86 ± 2.29 years. Mean BMI 26.2 ± 5.1. BMI class 1 (20 ≤ BMI ≤ 24.9) n=133, class 2 (25 ≤ BMI ≤ 29.9) n= 155, class 3 (30 ≤ BMI ≤34.9) n=53, class 4 (BMI ≥ 35) n=21, class V (BMI ≤ 19.9) n=34. Patient survival was not significantly different according to the recipient’s BMI class (p=0.476); graft survival was affected (p=0.031), as well as graft function up to 2 years post-transplant and at 4 years follow up (p=0.016). At logistic regression the factors independently associated with graft loss were only donor’s age (p=0.05) and BMI class of the recipient (p=0.002).

**Conclusions:**

Obesity did not impact on patient’s survival but affected graft function and graft loss.

## Introduction

Obesity represents a major healthcare alert worldwide with a growing incidence in the last decades, accounting more than 1.9 billion individuals aged > 18 years, being overweight (39% of the population), of which over 650 million obese (13%) (1).

High body mass index (BMI) poses critical consideration when selecting candidates for surgery (2). In particular, in view of the limited organ donor pool, there is still controversy whether end stage kidney disease (ESKD) patients suffering from obesity should be eligible candidates for the waiting list (3), or if given the increased risk of complications (4), mostly wound infections and dehiscence (5), but also delayed graft function and acute rejection (6), they should first lose weight as per a modifiable condition to optimize transplant outcomes (7).

Since kidney transplantation represents the best replacement therapy for ESKD (8), it could be seen as discrimination to not let a patient access this resource only because of his/her BMI status, especially if a living donor has come forward to avoid dialysis for the controversial candidate (9). Even for deceased donor transplantation there is increased life expectancy and quality of life in the literature well described (10), so ethically the decision to decline or delay a position on the transplant waiting list due to BMI alone cannot be taken lightly, but should be evaluated taking into account all the characteristics of the prospective recipient (11).

Additionally, while on the waiting-list, another important consideration must be given to the “obesity paradox” (12), a complex phaenomenon for which higher BMI is associated with improved outcomes and lower BMI with reduced survival. A possible explanation might consist in a better nutrition in general meaning a better immune response against chronic infections or other threatening complications, which are often a cause of death in the lower BMI dialysis population (13). This is also supported by the J-shaped association of dialysis mortality, where the nadir of the curve corresponds to normal BMI patients (14), while the historical unintended weight loss is an independent predictor of death (15). 

We have previously demonstrated that overweight and obese patients did not have inferior outcomes at one-year post-transplant (16) and that in the mid-follow up, i.e. 3 years, renal function, but not allograft survival was affected (16). The aim of the present study is to assess patient and graft outcomes of kidney transplant recipients in a steroid-free immunosuppression regimen at 5 years follow up, using BMI as a classifier.

## Patients and methods

This is an observational study of a single center kidney transplant recipient cohort who have consecutively undergone surgery between January 2014 and March 2016. Clinical data were prospectively stored in an electronic record. The primary outcomes were death-censored graft loss and patient survival. Graft loss was defined as need for return to chronic dialysis. Secondary outcomes included graft function, expressed as estimated glomerular filtration rate (eGFR) according to the Modification of Diet in Renal Disease (MDRD) (17) equation, measured at 3, 6, 12, 24, 36, 48, 60 and 72 months of follow up, as well as other factors known to be independently associated to graft loss.

Patients were stratified on the basis of their BMI calculated at the time of transplant as weight (in kg) divided by height (in meters) squared. In this way, the entire cohort was divided into 5 weight classes: group 1 = 20 ≤ BMI ≤ 24.9; category 2 = 25 ≤ BMI ≤ 29.9; class 3 = 30 ≤ BMI ≤34.9; group 4 = BMI ≥ 35 and category 5 = BMI ≤ 19.9.

All patients underwent treatment with a steroid-sparing immunosuppressive regimen (7-day course of steroids) with alemtuzumab induction and tacrolimus monotherapy (trough level, 5–8 ng/mL) or interleukin-2 induction with tacrolimus (trough level, 8–12 ng/mL) and mycophenolate mofetil.

The study was performed in accordance to the Declaration of Helsinki principles. The data used were anonymized and did not require patient or public involvement nor affected patient care. The study fell under the category of research through the use of anonymized data of existing databases which, based on the Health Research Authority criteria, does not require proportional or full ethics review and approval.

### Statistical analysis

Continuous variables are presented as mean ± standard deviation and compared using one-way ANOVA, ordinal and dichotomous variables with frequency and compared with chi square test. Survival was calculated with Kaplan-Meier estimate and the differences were evaluated with Cox regression. A linear regression model with backwards procedure tested which parameters are acting as independent predictors for graft loss. A generalized linear model of univariate repeated ANOVA with *post hoc* Bonferroni correction was used to determine whether mean eGFR differed statistically significantly among different BMI classes during follow up. Statistical analysis was performed using IBM*^®^* SPSS*^®^* Statistics version 27. The confidence interval was set to 95%, and p was considered significant at less than 0.05.

## Results

396 patients were included in the analysis. Donor’s and recipient’s demographic characteristics are reported in **Table 1**. At univariate analysis, only donor’s age was related to graft survival (p=0.002).

**Table 1:** Donor’s and recipient’s demographic characteristics.

	Characteristic	Overall	Graft survival yes	Graft survival no	OR (CI)	p
Donor’s	Age	51,8 ± 15,9	50.55 ± 15.9	56.64 ± 15.2	**-**	**0.002**
	Donation type (DBD/DCD)	195/81	156/61	39/20	1.43 (0.6- 3.2)	0.387
	CIT (hours)		13.69 ± 0.43	14.94 ± 0.75	–	0.151
	Type of donor •Cadaveric •live donor •simultaneous kidney-pancreas •pancreas after kidney	258 119 2 17	201 102 1 15	57 17 1 2	–	0.148
Recipient’s	Age	52,2 ± 12,8	51.77 ± 12.5	53.57 ± 13.8	–	0.268
	Sex (M)	260	215	45	1.46 (0.75-2.83)	0.137
	Ethnicity •Asian •Black •Caucasian •Mixed	134 39 157 66	110 28 132 52	24 11 25 14	0.73 (0.33-1.62) 0.45 (0.1-2.05) * 1.13 (0.46-2.76)	0.308
	BMI classes: •20 ≤ BMI ≤ 24.9 •25 ≤ BMI ≤ 29.9 •30 ≤ BMI ≤34.9 •BMI ≥ 35 •BMI ≤ 19.9	133 155 53 21 34	112 129 42 16 22	21 26 11 5 12	* 0.99 (0.54-1.82) 1.29 (0.58-2.86) 1.6 (0.53-4.81) 2.61 (1.14-5.99)	0.084

BMI, body mass index; CIT, cold ischemic time; DBD, donation after brainstem death; DCD, donation after circulatory death. For ethnicity, the comparisons are made between Caucasians and the other ethnicities. *For BMI, the comparisons are made between normal BMI (control group) and the other classes OR and CI are related to normal.

**Figure 1:**
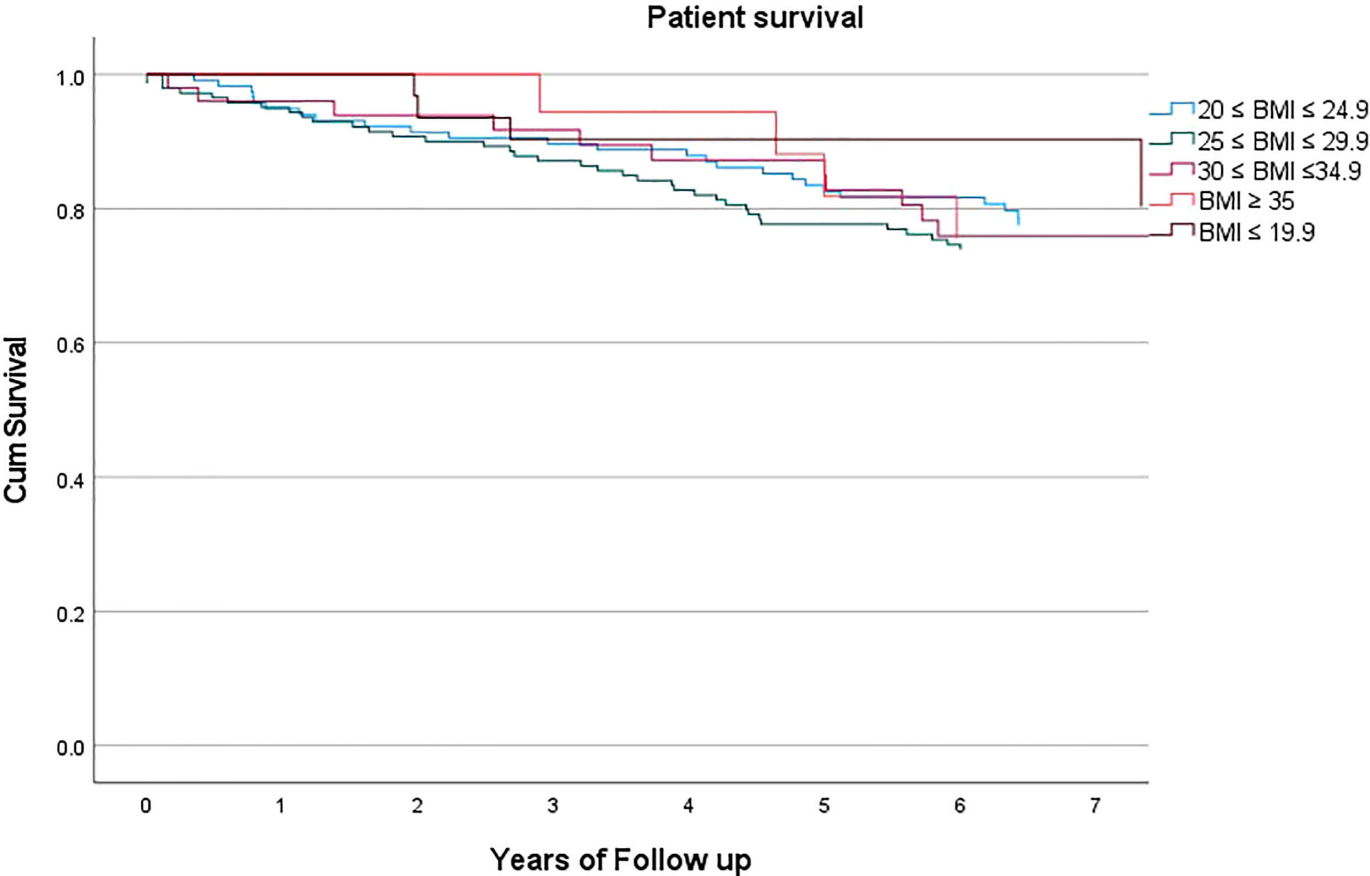
Patient survival according to BMI class.

Mean BMI was 26.2 ± 5.1. Mean follow up was 5.86 ± 2.29 years. Patient survival was not statistically significantly different according to the recipient’s BMI class (p=0.476, HR 0.935, C.I. 0.774-1.129), **Figure 1**. Expanding this further, at 5 years of follow up, mean patient survival was 77.5%, with 78.6%, 75.0%, 76.0%, 77.8%, 87.1% and 77.5% for class I-V respectively.

**Figure 2:**
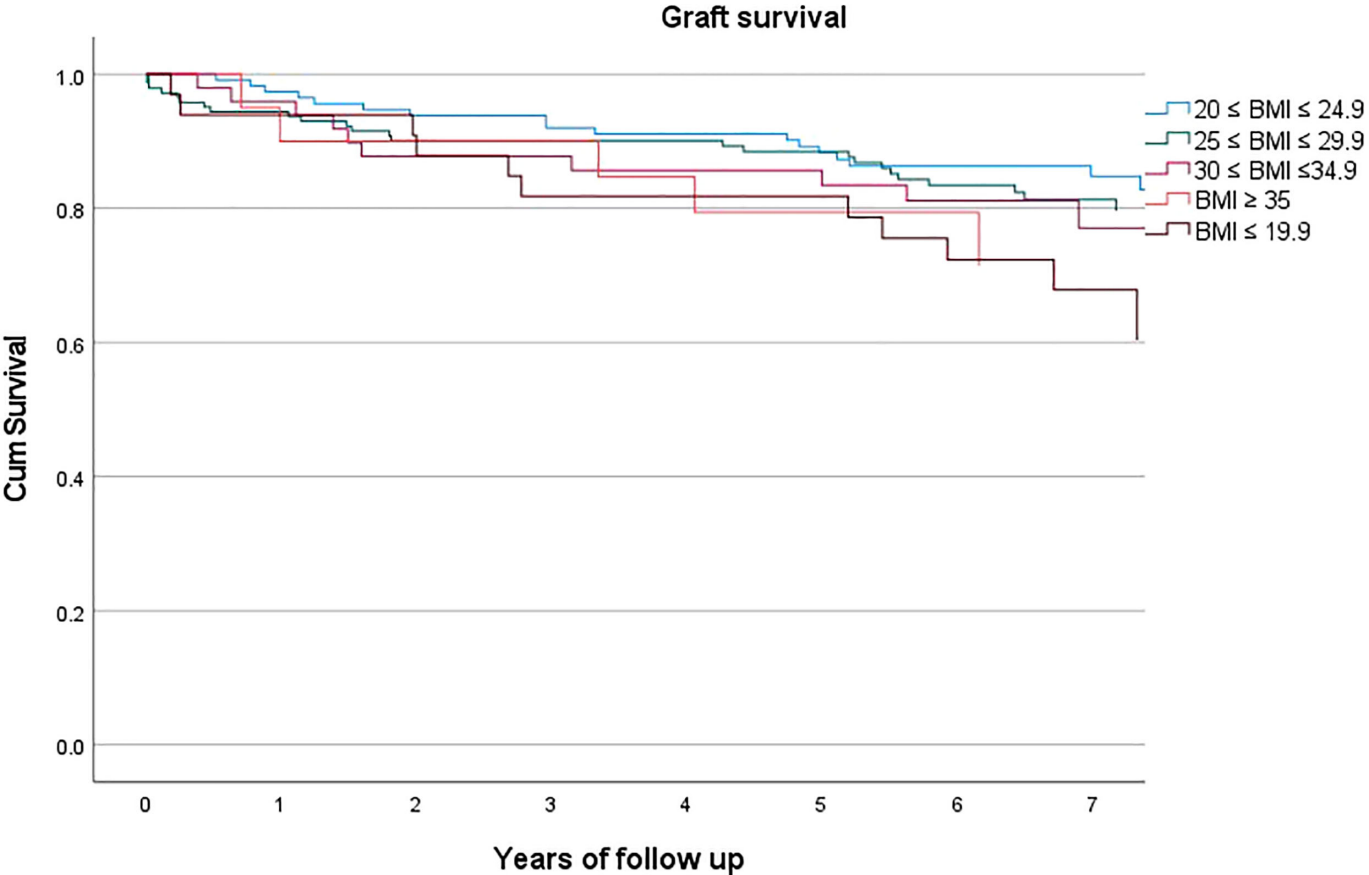
Graft survival according to BMI class.

However, graft survival was instead affected by BMI (p=0.031, HR 1.217, C.I. 1.024-1.448), **Figure 2**, with a mean survival of 80.3% and with 83.6%, 82.5%, 77.6%, 75.0%, 66.7% for class I-V respectively.

**Figure 3:**
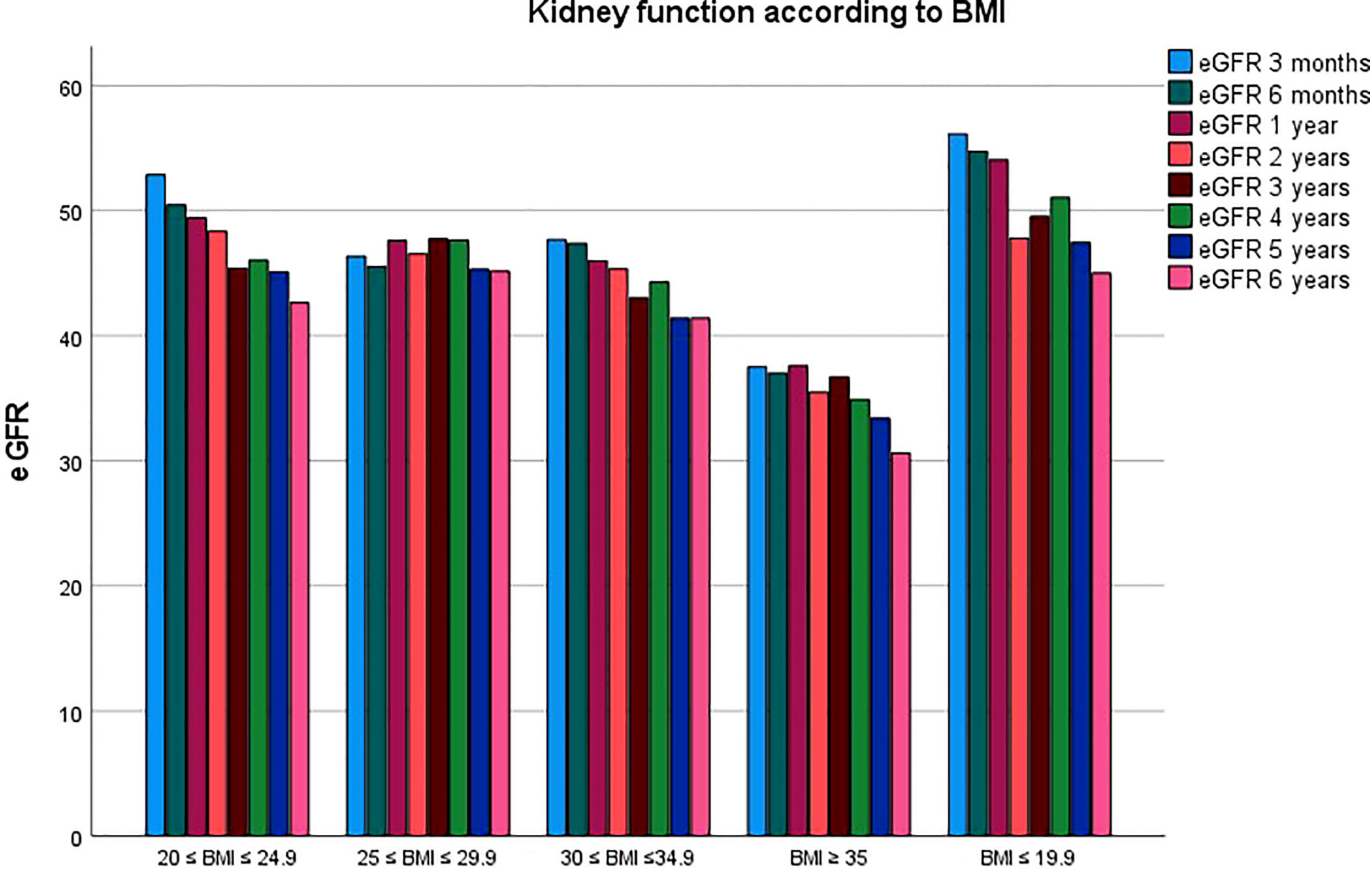
Comparison of kidney function between BMI classes.

Graft function was also significantly affected by BMI class during follow up. Results are summarized in **Table 2**, with a mean eGFR of 47.6 ± 19.32, 46.37 ± 18.99, 45.66 ± 17.3, 44.94 ± 17.52, 44.69 ± 17.27, 45.32 ± 17.12, 44.09 ± 18.62, 44.13 ± 18.86 ml/min/1.73m^2^ for all the classes at 3, 6, 12, 24, 36, 48, 60 and 72 months of follow up. **Figure 3** compares kidney function between the five BMI classes.

**Table 2:** Mean and standard deviation for kidney function during follow up per BMI category.

BMI category	eGFR 3 months p <0.001	eGFR 6 months p =0.001	eGFR 1 year p =0.026	eGFR 2 years p =0.009	eGFR 3 years p =0.211	eGFR 4 years p =0.016	eGFR 5 years p=0.496	eGFR 6 years p=0.187
* 20 ≤ BMI ≤ 24.9	52.49 ± 17.98	49.72 ± 17.97	47.79 ± 17.03	47.35 ± 17.56	45.27 ± 15.93	45.28 ± 15.25	45.59 ± 17.11	44.62 ± 18.14
25 ≤ BMI ≤ 29.9	44.46 ±17.89	43.17 ± 17.56	44.33 ± 16.29	43.28 ± 16.12	44.93 ± 17.02	46.14 ± 17.13	44.20 ± 18.44	44.87 ± 17.82
30 ≤ BMI ≤34.9	46.35 ± 20.71	46.31 ± 20.1	44.67 ± 18.82	43.44 ± 19.12	42.05 ± 17.98	43.74 ± 18.53	41.54 ± 19.32	42.87 ± 19.55
BMI ≥ 35	33.55 ± 13.91	35.47 ± 14.61	37.11 ± 15.72	38.06 ± 13.65	37.59 ± 15.5	33.53 ± 14.53	37.42 ± 15.31	30.60 ± 14.28
BMI ≤ 19.9	53.21 ± 22.26	53.52 ± 21.82	51.22 ± 17.56	48.65 ± 19.64	48.93 ± 20.55	52.07 ± 19.03	46.56 ± 23.26	47.12 ± 23.79
Total	47.61 ± 19.13	46.34 ± 18.86	45.76 ± 17.15	44.91 ± 17.37	44.65 ± 17.1	45.44 ± 17.06	44.25 ± 18.50	44.18 ± 18.79

Kidney function is expressed as eGFR ml/min/1.73 m^2^.

**Figure 4:**
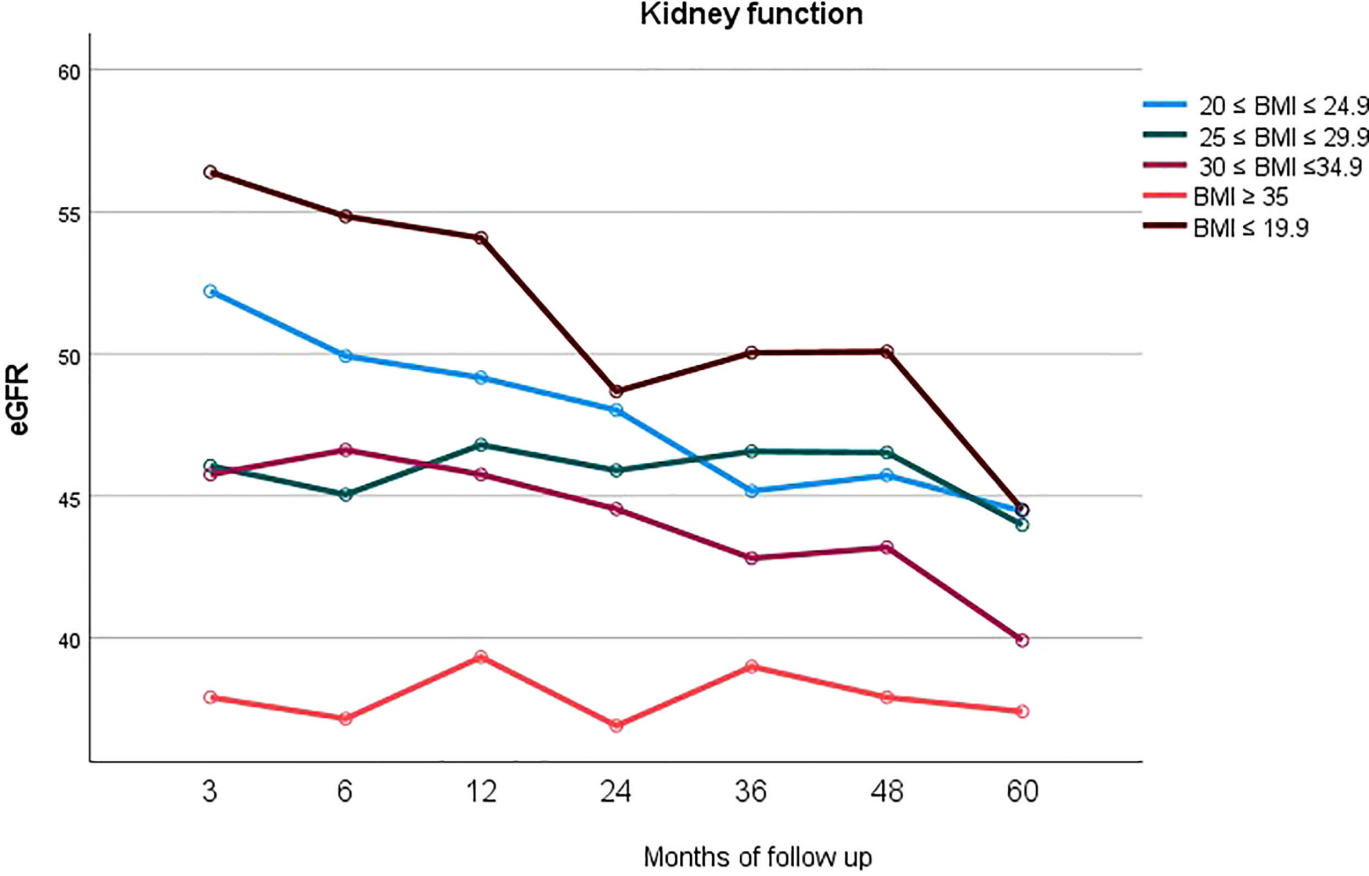
Longitudinal representation of mean eGFR up to 5 years follow up for the different BMI classes.

In **Figure 4** mean eGFR up to 5 years follow up for the different BMI classes is represented. Logistic regression showed that the factors independently associated with graft loss were only donor’s age (p=0.05) and BMI class of the recipient (p=0.002).

## Discussion

The survival benefit for end stage kidney disease following kidney transplantation over long-term dialysis is known (18), and in the present study we demonstrated there is no significant difference in terms of overall survival for patients, when considering BMI as a possible determinant. This poses the important question to allow access to a limited and precious resource, i.e., the organ donor pool, for patients otherwise discriminated only on the basis of weight and height (19). It is very easy to dismiss access for transplantation based on BMI, as an easily measured metric. However, this study shows that with a steroid sparing protocol at five years, overall survival probability is the same, independent of BMI, and thus the expected life after transplantation (20) is not a reason to not list patients based on BMI alone.

On the other side, a high BMI is proved to represent a risk factor for graft failure, and this confirms the necessity of a weight loss strategy ahead of transplantation (21), particularly in view of the incoming lifelong side effects of steroid therapy and immunosuppression (22). This concept is common to a more generalized pre-habilitation strategy and could be potentially translated also to other organs (23), but for kidney in particular, cardiovascular disease prevention and thus obesity treatment appears fundamental, given this represents a major cause of death (24). Pre-transplant diet and exercise should be encouraged before being actively listed for surgery, in order to improve aerobic and functional capacity, thus especially for frail candidates, as malnourished obese patients are, in order to reach a significant weight loss through a healthier lifestyle, including also diet and physical exercise. The issue for ESKD transplant candidates is yet the necessity of dialysis treatment while waiting for surgery, therefore the above described interventions may require years before becoming effective or could not even become a real option at all, while for the patient every more year spent on dialysis reduces the overall survival in a significant manner (25). Of note, we did not find inferior patient survival when stratifying for BMI classes, therefore the survival benefit kidney transplantation offers could be interpreted as uniform (26).

Bridge interventions, such as bariatric surgery are increasingly being adopted to overcome wait listing barriers and we support their utilization, particularly with regards to sleeve gastrectomy (27), given its lower risk of adverse renal consequences such as hyperoxaluria and nephrolithiasis (28), although there is still controversy on timings and on patient’s selection (3, 9). Additionally, potential post-surgical complications could translate into lower wait listing and transplant access for ESKD patients, therefore, there is still concern that bariatric surgery and the following weight loss could cause significant protein malnutrition and frailty, negatively impacting on dialysis patients, with worse waitlist and post-transplant outcomes (29).

From our analysis, there is evidence that graft function deteriorates over time and consequently leads to an increased risk of graft loss for obese individuals, as may other systemic diseases finally leading to end stage kidney function, particularly when recurring after transplantation (30). It is known in fact a direct causal connection between obesity and ESKD, because of an underpinning renal hyperfiltration driven by the excess weight, also known as obesity-related glomerulopathy (31). This syndrome can synergically act with other frequent comorbidities in obese patients, such as hypertension and diabetes, and in fact the latter was more common in higher BMI patients (p=0.0.22) (16). Furthermore, these conditions could worse in the post-transplant period, following immunosuppressive drugs administration, especially steroids (32). Yet, it appears not sustainable to condemn to lifelong dialysis someone only because of their BMI, but it is recommendable to rather intervening on modifiable risk factors for cardiovascular mortality, as for example to avoid steroids (32).

An interesting finding of our analysis is that donor age, and not donor type, affects the incidence of graft loss. Previous work reports that recipients of grafts from live donors aged < 60 have a 38% lower risk of developing acute rejection compared to those aged > 60 years (33). Since recipients of older grafts are generally also older in age, this leaves to the open debate on immunosuppression in the elderly, in whom, although physiological immunosenescence linked to biological aging is known, other potential contributors, such as the engraftment of older organs, is associated with higher rejection rates, and thus the need for tailored, age-adopted immunosuppression. If we attribute this to the fact that obese patients do suffer of immunosuppression side-effects more because of their intrinsic metabolic condition, it then could be favored the use of younger donors for obese recipients, especially because these candidates appear younger and fitter in general, to be selected for transplantation in view of their important comorbidities.

Finally, another important finding of the present study, is that graft and patient survival for class V (BMI<19.9) parallels those of class IV (BMI > 35). Although caution is warranted, given the sample size, we assumed that the obesity paradox might be the underpinning mechanism, in fact as already mentioned above, obesity in ESKD patients, may play a protective role (12) and could be associated with decreased mortality, particularly when looking at infections. Conversely, the presence of signs of undernutrition, like BMI <19.9, that is often associated to frailty, could lead to a higher susceptibility to serious complications (34), such as sepsis, another major cause of mortality in transplanted patients.

Our study presents some limitations: patients fit for transplantation were selected and many have undergone intensive medical workup to optimize their cardiovascular risk factors. This could have biased in selection for transplantable patients, especially in the high BMI cohort. Also, the use of BMI as a measure for adiposity is imperfect because it does not differentiate between fat and lean body mass, as could girth, for example, although most population variance in obesity is explained by BMI.

In conclusion, the present study suggests that obesity is not an absolute criterion to exclude a patient from the kidney transplant waiting list. Further research is warranted to investigate whether another surrogate marker for obesity could be adopted, and which patients might benefit of an overall bariatric strategy. If the possibility of a living donor comes forward, the transplant should not be postponed, as a survival benefit over dialysis is to be preferred to the risks related to the decision to defer it.

## Data availability statement

The original contributions presented in the study are included in the article/supplementary material. Further inquiries can be directed to the corresponding author.

## Ethics statement

The study was performed in accordance to the Declaration of Helsinki principles. The data used were anonymized and did not require patient or public involvement nor affected patient care. The study fell under the category of research through the use of anonymized data of existing databases which, based on the Health Research Authority criteria, does not require proportional or full ethics review and approval.

## Author contributions

Conceptualization, MB and PH; methodology, MB, ED, FC, and PH; validation, MB, FC, and PH; formal analysis, MB and FC; data curation, MB, ED, and PH; writing—original draft preparation, MB; writing—review and editing, MB, FC, and PH. All authors contributed to the article and approved the submitted version.
